# Harnessing of Social Capital as a Determinant for Climate Change Adaptation in Mazungunye Communal Lands in Bikita, Zimbabwe

**DOI:** 10.1155/2021/8416410

**Published:** 2021-04-19

**Authors:** Louis Nyahunda, Happy Mathew Tirivangasi

**Affiliations:** Department of Research Administration and Development, University of Limpopo, P.bag X1106, Mankweng 0727, South Africa

## Abstract

The livelihoods of rural people have been plagued by the precarious impacts of climate change–related disasters manifesting through floods, heat waves, droughts, cyclones, and erratic temperatures. However, they have not remained passive victims to these impacts. In light of this, rural people are on record of employing a plethora of adaptation strategies to cushion their livelihoods from climate change impacts. In this vew, the role of social capital as a determinant of climate change adaptation is underexplored. Little attention has been paid to how social capital fostered through trust and cooperation amongst rural households and communities is essential for climate change adaptation. This study explored how people in Mazungunye communal lands are embracing social capital to adapt to climate change impacts. The researchers adopted a qualitative research approach guided by the descriptive research design. The population of the study was gathered through simple random and purposive sampling techniques. Accordingly, the population sample consisted of 25 research participants drawn from members of the community following the simple random and purposive sampling techniques. In-depth individual interviews and focus group discussions were used to collect data. Data were analysed through the Thematic Content Analysis. This study established that different forms of social capital are being embraced by the community members to withstand the effects of climate change. These include village savings clubs (*fushai*), chief's granary (*Zunde raMambo),* collective field work (*nhimbe*), and destocking of livestock (*kuronzera*) strategies. These strategies illustrate community reliance on indigenous knowledge adaptation strategies as a community response to impacts of climate change on their livelihoods.

## 1. Introduction

Climate change adaptation refers to response strategies employed by human or natural systems to reduce exposure to climate change risks and to minimise harm or exploit the benefits that come with adaptation [1]. As such, adapting to climate change involves employing mechanisms that minimise the effects posed by climate change, making use of the progressive mechanisms and initiating changes respectively [2–5]. In the face of climate change impacts, adaptation strategies are invented to modify or secure livelihoods. This is because climate change ravages lives and livelihoods with its impacts highly prevalent in rural communities where there is high reliance on climate volatile resources for survival [1, 6]. Considering this, Hinkel [7] opines that the purpose of adaptation is for social-ecological systems (rural people) to organize itself and respond to climatic induced damages, learn from them, and make use of the advantages present to moderate the risk.

In response to the above, Yohe and Tol [8] denote that adaptive capacity has its own determinants that when present in any system, it can be prudent to assert that individuals, communities, and institutions are adapting to climate change; the availability of technological advancements enables adaptation, the equal distribution of resources, availability of human and social capital including literacy levels, and social and personal security and how the public perceive the cause of the disturbances (climate change) and its manifestations. At this juncture, the researchers paid attention to social capital as a determinant of adaptation to climate change. This is because other components of adaptive capacity require advanced resources unlike social capital which is embedded in social networks. In corroboration, Chagutah [9] notes that relevant technologies for adaptation are not easily accessible for rural people due to their high cost, lack of skills complemented by illiteracy, and unavailability of early disaster warning dissemination systems. Nevertheless, [10] avowed that the determinant of adaptive capacity which is available in most rural communities is the stock of social capital which refers to a social web of networks created through interaction which strengthens trust and social norms.

In the same line of argument, Musavengane and Simatele [11] contend that social capital has a long-embedded record of playing a role in individual, household, and community risk smoothing. On the same note, the significance of social capital manifests through sharing of best practices and postdisaster recoveries which are vital components for adaptive capacity. Against this backdrop, this study investigates how social capital is fostering adaptation to climate change in Mazungunye communal lands, Zimbabwe. As for Nyahunda and Tirivangasi [1], the precarious impacts of climate change are visible in Mazungunye community manifesting through recurrent episodes of drought, death of livestock, erratic rainfall patterns, dwindled crop production, and heat waves. Inspite of all this, rural people in Mazungunye communal lands have not remained passive victims of climate change impacts, but they have responded to its vagary impacts through a cocktail of adaptation strategies either individually or collectively [10]. In response, Apgar et al. [12] aver that several studies on climate change adaptation have focused on other transactional forms of adaptation. However, little attention has been paid to how social capital fostered through trust and cooperation amongst rural households and communities is essential for climate change adaptation. As such, this study sought to close that knowledge gap.

### 1.1. Theoretical Framework

The Social Capital Theory guided this study. The theory connotes that relationships matter in every society because they foster cooperation, trust, and reciprocity that can positively impact the wealth of the society through reduction of transactional costs, curbing of opportunistic behaviours, and facilitation of collective actions [13]. In the broader view of Social Capital Theory, Aldrich and Meyer [14] opine that there is a prediction that higher associational activities inside a community foster a sense of civic engagement where mutual trust, cooperation, and reciprocity are developed and used to solve collective action and other pressing problems. In addition, Scheffran et al. [15] avowed that connections and social obligations within members of a group constitute social capital. These can be connections through friends, kinship, and neighbours where information, labour, and resources are shared in adaptation strategies. As for Petzold and Ratter [16], the Social Capital Theory views social capital as a confluence of various entities sharing the common characteristics of facilitating certain actions of individuals within the structure and organisation of some aspects of a social structure.

To add on, this implies that social capital is defined by its function. As such, the importance of social capital is hinged by the interactions between members [17]. In light of this, the researchers aver that the Social Capital Theory lenses were critical in exploring the interplay of relationships, norms, and social networks in fostering adaptation to climate change in Mazungunye communal lands. Considering this, Gooderham et al. [18] contend that human capital resides in individuals, whereas social capital resides in relationships and trust is a fundamental ingredient of the engine that makes these relationships work. Also, social capital is anchored by social networks and these networks describe how and whom individuals interact including the exchanging of information or resources [19]. Stafford and Hartman [20] added by alluding that social networks devise strategies in preparation, and these networks facilitate physical, emotional, and financial support during an event. Furthermore, norms, trustworthiness, and networks that tie people together define social capital. In this regard, social capital can manifest through practical means and initiatives where important tasks are accomplished [21]. Through social capital, Jordan [22] opines that members within the social network may converge for gatherings be it for entertainment but later for some constructive purposes. Lastly, Rodima-Taylor [23] mentions that unlike other forms capital, social capital is much more democratically distributed, and this becomes a powerful engine of social mobility where it sanctions effective norms and information channels between members who share similar values and norms.

### 1.2. Social Capital and Climate Change Adaptation

Adaptation to climate change rests on a social component as individuals interact with other network members to share resources, gain information, build new institutions, and create collective norms, in order to provide resilience to climate change. As such, social capital serves as a public good in supporting whole communities through extreme events such as climate change [5, 24]. In corroboration with this, social capital can contribute to how smallholder farmers respond and adapt to climate change and assist to ensure food security and the resilience of livelihoods. Furthermore, social capital has the potential to enhance people's livelihoods and transfer of knowledge and information among people [25]. In rural communities, social capital is very important and there is high reliance on it for cooperation and cooperatives aimed to foster alternative responses to external shocks such as climate change [26]. To add on, social capital is one of the many resources available to individuals within a community; therefore, its effectiveness in fostering adaptation to climate change can be widely acknowledged by most community members [27].

The significance of social capital is evident during climate-induced shocks where individuals with strong social ties to neighbours, kinships, and friends are more likely to return and restore a damaged neighbourhood and their livelihoods [28]. Most importantly, social capital facilitates a bottom-up coordinated response which is inclusive of local components share across the society [19]. In the same wavelength, Aldrich and Meyer [14] argue that social capital fosters community engagement, cooperation, and participation which are essential in addressing community challenges such as climate change. Similar sentiments are shared by Hinkel [7] that adaptation to climate change is enhanced through embracing social capital where social networks facilitate the adoption of viable agricultural technologies; act as a mediator for financial transfers that may ease farmers' credit constraints; and provide information on new technologies and facilitation of cooperation among farmers to allow the different dimensions of social capital, which include sharing of information and scarce resources [10]. Lastly, Musavengane and Simatele [11] posit that social capital can be a vehicle through which the accumulation of different forms of capital can be achieved and contribute to environmental management. Reasoning from the above literature, the researchers argue that social capital can be a strong vehicle in fostering a sense of togetherness and community cohesion where social networks provide a unique adaptation resource to rural people who do not have access to other forms of adaptation that require technological advancements.

## 2. Materials and Methods

### 2.1. Description of the Study Area

**Figure 1:**
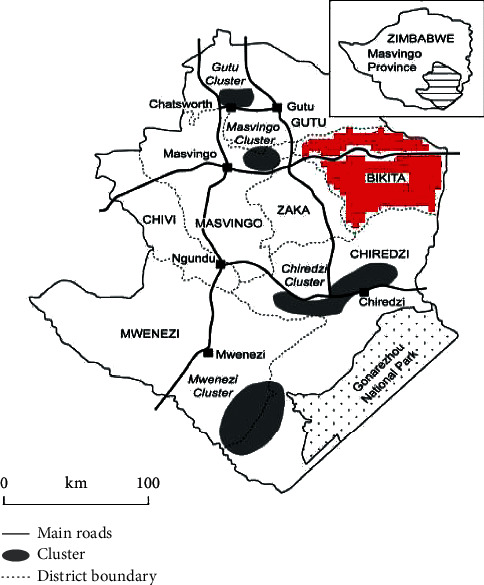
Map of Zimbabwe showing Bikita district (in red) where Mazungunye community is located.

The Mazungunye communal lands is found in ward 4, Bikita district of Masvingo province in Zimbabwe as shown in Figure 1. Masvingo province is in the south-eastern part of Zimbabwe. Bikita is approximately 80 km east of Masvingo Urban. Bikita district (highlighted in red in Figure 1) covers an area of 10, 000 square kilometres and has a population of about 80, 000 people. Mazungunye communal lands lie in Zimbabwean Agroecological Region 4. This is a semi-intensive farming region experiencing a mean annual rainfall between 300 and 600 mm with a 40%–45% coefficient of variation. It is subject to periodic seasonal droughts and prolonged dry spells during the rainy season [1, 29, 30]. The study area is made up of four villages, namely, Chiwawa, Jere, Pfunde, and Maipise. The majority of people in the study area are peasant farmers practicing farming based on growing crops and livestock rearing. These communities have been riddled by climate change impacts for some time because of the climate volatile ecological region where they are situated [1].

### 2.2. Research Approach

This study was qualitative in nature guided by the descriptive research designs. Considering this Lune and Berg [31] denote that qualitative methods yield data about human views over subject matter of concern, that is, the often-contradictory behaviours, relationships of individuals, beliefs, emotions, and opinions. As for Sekaran and Bougie [32], the use of the descriptive design provides an elucidation of salient details of a situation be it in a social setting or relationship. On the same note, [33], descriptive research design involves observations and description of the behaviour of a subject under investigation without any form of influence by the researcher. As such, the advantage of the descriptive research design is that subjects are observed in their natural setting where their natural environment is unchanged.

### 2.3. Population and Sample Size

The study population was drawn from the members in Mazungunye communal lands. Accordingly, the simple random technique was adopted to select the six villages. In the same process, the purposive sampling was followed in the selection of the study participants within these villages. Simple random sampling warrants that each individual case in the population has an equal chance of being selected in the study sample [34]. On the same note, purposive sampling allows the selection of participants according to the needs of the study and the judgement of the researcher that the identified participants can provide the required information [35]. Guided by these sampling techniques, the researchers selected (25) twenty-five participants distributed as twenty (20) ordinary community members and five (5) traditional leaders. These participants were selected on the presumption that they are practicing subsistence farming which is a common livelihood activity in Bikita district [30] and that they are experiencing the effects of climate change which necessitates the usage of social capital as an adaptation strategy.

### 2.4. Data Collection and Analysis

Focus group discussions and unstructured in-depth individual interviews were used to solicit for information from the participants. Accordingly, focus group discussions were used on ordinary community members brought together to share their experiences with climate change and how they are embracing social capital to adapt to its impacts. Individual interviews were used on traditional leaders who served as key informants. This was done under the presumption that some societal norms and values which enhance social capital are sanctioned by traditional leaders. As such, the researchers held one focus group discussion in each village where each meeting comprised of five participants. One individual interview was conducted per village with traditional leaders. Shona was used as the medium of communication through which the participants could share their views. The proceedings were audio taped. The Thematic Content Analysis was followed to analyse the data from which the findings were drawn. When using the thematic content analysis, the researchers started with data transcription followed by the selection of transcripts and compilation of themes. The process further involved the categorization of themes, recoding of data, and the writing up of the manuscript.

## 3. Results and Discussion

The researchers documented activities undertaken by community members of Mazungunye communal lands which point to the manifestation of social capital in adapting to the impacts posed by climate change. What should be underscored is that these activities are fostered by relationships and social networks where collective efforts are made to address common problems and improve human wellbeing. That being said, this study found that in Mazungunye communal lands, social capital as an adaptation to climate change impacts is embraced through collective field work (nhimbe), chief's granary (Zunde raMambo), village savings clubs (Fushai), and destocking of livestock (kuronzera).

### 3.1. Collective Field Work (Nhimbe/Humwe)

Nhimbe is a strategy that involves mutual aid through collective sharing of resources by members of a community, which is an indigenous move towards alleviation of environmental problems such as drought. As such, the nhimbe strategy is practiced on many tasks such as ploughing, planting, weeding, harvesting, construction, or threshing. The guests for *nhimbe* come with necessary resources such as labour, draught power, ploughs, hoes, and expertise depending on the nature of the task. Under this strategy, social capital is manifesting through community members collaborating in their agricultural activities and then later drink traditional beer. Also, the host of the nhimbe contributes the food and drinks such as traditional beer (*ndari*) and *mahewu* (a form of traditional beer without alcohol content). A significant proportion of participants indicated that the nhimbe strategy is about enhancing food security in the community amidst climate change threats. It emerged in this study that the *nhimbe* strategy is meant to strengthen the social nets of the community against the risks posed by climatic events such as droughts and floods. Through the nhimbe practice, community members converge to share skills, expertise, ideas and experiences about how to address drought and information about viable farming methods. From this, the researchers infer that this adaptation strategy connotes that mammoth tasks can be executed in a short space of time through collective efforts. The advantage of this strategy is that given the erratic rainy seasons brought about climate change, community members can easily perform tasks before dry spells hit the grounds especially those dedicated to ploughing and planting. The following statements confirm this.

“…We believe that one man's finger cant crush lies (*chara chimwe hachitswanyi inda*) so when one of us has a mammoth task to be executed in the fields, he/she can request for labour from the community members and we are always ready to help another because *kandiro kanopfumba kunobva kamwe* (a hand that gives is the hand that receives) especially in trying times like these…” (FGD 1)

“…Instead of looking for help from outside where one must pay for labour, we call for *humwe* to help each other with any task at hand. We learn a lot from these collective gatherings and we also achieve a lot especially in times of drought, we have to learn from each other on how to boost our food production systems …” (FGD 2).

The above narrations give credence to Tenzin et al. [25] who avowed that social capital has the potential to enhance people's livelihoods and transfer of knowledge and information among people. The following narration provides further insight.

“…The hosting of *nhimbe* brings us together as a community to fight the enemy called *nzara* (drought). By so doing we are accustomed to planting small grain crops like raphoko (*rukweza*), sorghum (*mapfunde*) and millet (*mhunga*). These crops have a cultural significance for brewing of beer we drink at nhimbe and during our rain making ceremonies (*mukwerere*) where we ask for rains from our ancestors. Small grain crops are also viable to withstand the long dry spells which are facing…” (Traditional leader, 2).

From the above, the researchers argue that the promotion of small grain crops for the use of beer brewing, food security, and other cultural significance points to how social capital is enhancing crop diversification which has been reported by various studies to be a common adaptation strategy among rural people in Zimbabwe [10, 36–38]. What should be highlighted is that the *nhimbe* strategy is serving as a platform for networking, sharing of skills, and problem-solving knowledge. Most importantly, it serves as a matchmaking practice where the morale of the guests is boosted through eating and drinking together. From this, the researchers argue that this strategy bears advantages for the community members in the wake of climate change setbacks because of shared responsibilities and information about viable farming methods. The collective execution of tasks brings a modicum of assurance that social capital harnessed through the *nhimbe* practices transcends to protection and support of each other even beyond climatic perturbations. This serves as a baseline for resilience in Mazungunye communal lands. To add on, the participants indicated that the nhimbe practice builds cooperation, trust, solidarity, allegiance, and respect which are key proponents in creating safety nets against the burgeoning climate change impacts. It is worth mentioning that these are the proponents of social capital as outlined by Collins, Neal and Neal [21] that norms, trustworthiness, and networks that tie people together define social capital. In that regard, social capital can manifest through practical means and initiatives where important tasks are accomplished. The following narration provides more insight.

“…. These unpredictable weather changes require us to be united and come up with collective solutions because if you cannot mourn with others, no one will mourn with you when you are trapped by disasters….” (FGD, 3).

From the above, the researchers exposit that the collective pool of social capital is necessary to build community resilience against climate-induced shocks like drought. This gives credence to Chanza [4] who avers that social capital tends to influence the success of climate interventions targeting local communities. As such, the collective community participation, problem solving, sharing of experiences, and networking through the nhimbe strategy provide solutions for disaster management which speaks to the role of social capital as stated by Aldrich and Meyer [14] that social capital fosters community engagement, cooperation, problem solving, and participation which are essential in addressing community challenges such as climate change. In support of the above, Thamaga-Chitja and Tamako [39] opine that adaptation to climate change is created by a social component, interacting with others, networking to gain information, the sharing of resources, and creating collective norms to build resilience against climate change. Based on this, the researchers contend that the *nhimbe* strategy has some shortfalls to be noted. In this regard, it is noteworthy mentioning that only those with resources to host the guests for the tasks to be executed under *nhimbe* can afford to do so. What this means is that community members who cannot afford to provide for food and drinks needed to host the *nhimbe* are not reaping the benefits of this strategy. This is because this study could not establish whether community members make contributions for each other to host the *nhimbe* apart from the help they provide for the specific tasks to be executed. Another weakness of the strategy would be in the form of it requiring only those who are physically fit to execute the tasks at hand. Also, this strategy is not viable during health emergencies such as the Corona virus disease (COVID-19) pandemic where contact-based activities are prohibited.

### 3.2. Chief's Granary (Zunde raMambo)

Zunde is a Shona word that may mean a large gathering of people taking part in a common activity or may refer to plenty of grain stored for future use by people in a community [4]. As for Gukurume [10], Zunde raMambo is a Shona phrase which means “the Chief's granary.” The Zunde is a common field designated by a chief that is Chief Mazungunye for cultivating food crops by the community. Under the rural governance systems, the village head (Sabhuku/Sadunhu) shoulders the responsibility of ensuring that village members participate in the Zunde fields as per the routines outlined by the Chief. Participation in the Zunde is compulsory for every household and defiance to take part is regarded as a taboo and bears punishment by the Chief. The harvest from the Zunde is stored in a common granary under the direction of the chief. This granary is built collectively by the community members as a reservoir for the agricultural proceeds. As such, this study noted that the Zunde granary has the carrying capacity of approximately 500 tonnes of maize grains in a season cycle. Also, there are other smaller granaries built adjacent to the main granary for the storage of small grains proceeds such as millet (*mapfunde*), rapoko (*zviyo*), and sorghum (*mhunga*). These can store up to 100 tonnes of grains each. The primary aim of the Zunde is to ensure that a community has food reserves which could be used in times of food shortages [40]. From this, the Zunde granary can store up the grains in the span of two to three years or more depending on how the stocks are treated from pesticides. The granaries are erected at climate resistant places such as on rock tops and above the ground sustained by strong rock corrugations.

In light of the above, this study found that in times of agricultural catastrophes owing to climate change the “Zunde raMambo” system serves as a local social safety net for the poor and vulnerable members of the community. The respect for norms and values that sanction the compulsory participation in the field designed to provide safety nets for the community speaks to the proponents of social capital as avowed by Bernier and Meinzen-Dick [19] that it sanctions effective norms by encouraging the adoption of different types of practices such as distribution of resources, sharing of information, and contribution to different outcomes such as the Zunde strategy. What should be highlighted is that the Zunde raMambo practice serves as a safety net against drought for the community. The following narration confirms this.

“…The Zunde raMambo is the integral part of our culture and we are obliged to participate in it to prepare for future misfortunes like drought (*zviuya zvemberi senzara*). The Zunde harvests help us a lot that's why we have so much respect for it and we share ideas collectively to improve production….” (Traditional leader, 3).

In Mazungunye communal lands, Zunde is perceived as a social, economic, and political rallying mechanism apart from it being a crop production activity whose main objective is to address food shortages. This gives credence to [39] who posit that social capital can contribute to how smallholder farmers respond and adapt to climate change, assist to ensure food security, and the resilience of livelihoods.Towards this end, in Mazungunye communal lands, participation in the Zunde raMambo is an expression of oneness and carried with social and moral obligations. As such, the proceeds from the Zunde are perceived as a social tool which brings people together to share their successes and/or failures. This is in tandem with the Social Capital Theory which connotes that social obligations within members of a group provide a platform for shared labour and resources for adaptation [15]. In Mazungunye communal lands the purpose of the Zunde practice is to ensure that food security is guaranteed all the times; hence, the obligation to participate in the fields is voluntarily shared by the community members. The following statement confirms this.

“…We benefit a lot from the Zunde raMambo. We know that it is the granary for orphans (*dura renherera*) where we find rescue during drought therefore, we participate with high spirits….” (FGD, 4).

Extrapolating from the above, the researchers established that the Zunde raMambo practice, which is a traditional in-built mechanisms of adapting to livelihood shocks in the community, embraces social capital to boost productivity. The Zunde raMambo provides food portions for village ceremonies such as funerals. In the same line of argument, traditional leaders emphasised that they implore the practice to be strengthened because it is their shared project poised to increase food production with the intention of evading the high dependence on external food security interventions. Traditional leaders appreciated the Zunde practice as a security mechanism designed to address the contingency of drought and famine. The following narration confirms this:

“…. We acknowledge that our fields are no longer producing enough food to take us through the year however we dedicate our collective efforts in the Zunde raMambo because we know that there will be democracy in the distribution of food when need arises unlike with these donor food parcels….” (Traditional leader, 1).

The above narration gives credence to Aldrich and Meyer [14] who avowed that, unlike other forms capital, social capital is much more democratically distributed, and this becomes a powerful engine of social mobility. In the same line of argument, Thamaga-Chitja and Tamako [39] postulate that social capital is one of the many resources available to individuals within a community. In this regard, the researchers aver that this democratic component of social capital is manifesting through just distribution of the proceeds from the Zunde raMambo which makes adaptation to climate change palatable for the inhabitants of Mazungunye communal lands. To add on, Chanza [4] opines that the Zunde raMambo practice deserves emphasis against climate change risks and the weakening of national social security schemes attributed to resource constraints, erratic food relief supply chains, and politicisation of food aid. Reasoning from the above, the researchers argue that the major advantage of the Zunde strategy is that it serves as proactive measures to avert the conundrums of hunger owing to climate change impacts. This is because the national granary, the Grain Marketing Board of Zimbabwe, has been negating the need for providing maize grains to the hungry in recent years [30]. However, one of the weaknesses of the Zunde strategy is that it is still depended on rain-fed agricultural activities. So, when the communities receive low rainfall to boost agricultural production, it means there are no proceeds to stock up in the Zunde granaries. At the end of the day, these communities end up being vulnerable to drought because the Zunde granaries get dried owing to the recurrent episodes of failed harvests.

### 3.3. Village Savings Clubs (Fushai)

The term *Fushai* is the Shona translation of how rural people dry vegetables (*mufushwa*) for future consumption. In light of this, the process of savings clubs is equated to a process where money is invested in a group fund for future use. In Mazungunye community, the participants reported to have diversified their livelihood strategies to scale up the income portfolios as a panacea to withstand the livelihood shocks dovetailed by climate change. This study established that social capital is manifesting through the *Fushai* practices which is one of the off-farm activities embraced by the community members to improve their sources of income guided by officials from CARE International, one of the nongovernmental organisations operating in the area. The Fushai project is a community-based, self-managed village savings groups to enable poor households to better withstand shocks and stresses.

Members of the savings club organize themselves into groups and agree on a monthly contribution per member towards the group fund. The combined savings create an internal loan scheme where members can borrow money from the internal fund and reimburse the money at an agreed interest. The savings pool continues to grow through monthly contributions and revenue from interest rates charged on internal loans. Accordingly, there is an agreed amount of money accumulated that will go to a different member after every two weeks guided by the operation cycle. Depending on how the fund grows, the loans will be given to several members at once. The size of the group determines the duration when all members can receive a loan. When the operating cycle comes to an end, the group shares out the total value of their loan fund among members.

What should be highlighted is that social capital is manifesting in the Fushai practice because the groups are formed on kinships, neighbourhood, and friendship hinged on trust and mutual dependence. The level of trust is evident in the commitment of the club members that no member defaults the contribution or in reimbursing the loan. All group members adhere to the basic set of principles. The Fushai project has no external borrowing or donations; hence, it enhances ownership and use of community resources. The researchers regard this practice as an adaptation strategy to climate change evolving through harnessing of social capital. This is because the benefits of the Fushai practice are sought to fight the impacts of climate change in the communities. The following narration confirms this:

“Our fields are no longer producing enough to sustain us, so we are grateful for the *Fushai* project. We are now able to supplement our incomes and support our households. As such, we are no longer vulnerable to drought because we get money to secure food and other assets from Fushai…” (FGD, 5).

In the four villages where the *Fushai* project is dominant, participants reported an increase in household assets where they amassed money to buy agricultural inputs, small livestock that are drought tolerant, construction of quality houses that can withstand floods, drilling of wells to curb water shortages and supplementary income to pay for school fees and health care bills. A study conducted by Thamaga-Chitja and Tamako [39] revealed that through savings clubs, members converge for meetings to share ideas and problems, sharing resources and draught power strengthening social networks and social capital. According to Mugiya and Hofisi [40], savings clubs help farmers to diversify and invest in less climate sensitive ventures. In addition, it offers a platform to drought exposed farmers to strengthen their financial management attributes. This further gives credence to Chanza [4] who avers that social capital is very important in rural communities because people rely on cooperating and cooperatives for alternative responses to external shocks such as climate change. The researchers argue that the *Fushai* practice promotes asset acquisition and asset-based savings which is a symbol of household wellbeing essential for fostering adaptation to climate change impacts. With that being the case, the researchers also noted that the strategy has some pitfalls attributed to the lack of legal back up and remedies to insulate the members in the event other group members defaulted or when the borrowers fail to reimburse the money. Another pitfall is that the stokvel money is not kept at the banks for security purposes, so under circumstances where the custodians of the money (group leaders) get subjected to robberies or other eventualities that might lead them to lose the money, the whole process may crumble without means of leaping back.

### 3.4. Destocking of Livestock through Lending to Relatives (Kuronzera)

In Mazungunye communal lands, climate change impacts on agriculture are evident in crop failure, desiccation of water points, drying of pastures, and death of livestock. Furthermore, climate change is aggravating the situation where erratic temperatures are causing high incidences of diseases for livestock. Rains have been erratic and more unpredictable and the main rainy season has been characterised by trends of irregularities. Cattle production under communal rangelands in Zimbabwe is constrained by a myriad of challenges including feed and water shortages, parasites, and diseases [41]. Several studies on climate change impacts reveal that most smallholder farmers have been experiencing colossal loses of livestock owing to climate change. As such, these farmers have resorted to destocking their livestock particularly cattle through selling or slaughtering for consumption [10, 36, 37]. What emerged from this study is that the forms of destocking through selling or slaughtering of livestock is not viable among the participants since it serves as a backlash to the economic status of rural people. In light of this, the participants submitted that they embrace social capital by lending their livestock to their relatives and friends located where there are better grazing lands through the system called *kuronzera*. The following narrations confirm this:

“We do not have control over drought but slaughtering of livestock is not a viable option. Cattle define a man. We can only sell in times of emergency. We thank God that other parts of Bikita receive better rains and some are irrigated therefore we take our livestock to our friends and relatives to spare them from dying…” (FGD, 6).

“… *Zvanza zvinogezana muno mwanangu* (a hand washes another hand), we acknowledge that drought is killing our livestock because the pastures are bad. Killing them is not the option because we use them for draught power in farming, so we normally drive them to our loved ones where the pastures are good…” (FGD, 3).

Extrapolating from the above, the *kuronzera* system is regarded as the most effective practice because the owners would not lose their valuable asserts. The importance of social capital is evident where people capitalise on relationships and social networks to rescue one another from disasters. What also emerged is that even community members who are not directly related to those in better grazing areas make use of their neighbourhood relationships to drive their livestock in those areas as a result of social networks created. This validates the proponents of social capital outlined by Stafford and Hartman [20] that social networks facilitate the adoption of alternative adaptation strategies which include sharing of information and scarce resources. The researchers argue that the insulation of livestock from drought through lending to relatives is a food security mechanism because livestock serves as means of farming, milk, meat, and a source of financial income. Another strength of the strategy is that it saves the livestock as compared to destocking through slaughtering. To the contrary, it is worthy mentioning that this strategy is not sustainable for one good reason that is the universality of climate change impacts across the district. As such, all communities are likely to experience high temperatures and erratic rainfalls that may lead to the depletion of grazing lands for livestock. Owing to lack of irrigation facilities in most parts of Bikita district, this strategy makes efforts to redeem livestock from drought a futile exercise.

## 4. Conclusions

This study established that in the face of climate change impacts, the inhabitants of Mazungunye communal lands are embracing social capital among other adaptation strategies. Social capital is widely embraced in the communities because it is readily available, and its proponents can be easily shared and owned by people in finding common solutions to problems stemming from climate change perturbations. What should be highlighted is that harnessing of social capital as a determinant for adaptation to climate change impacts does not imply that rural people are not confronted by challenges in the process. Like other rural communities in Zimbabwe, people of Mazungunye communal lands lack adaptive capacity to climate change impacts aggravated by lack of technologies, strong institutions essential for risk and disaster reduction, low educational levels, poverty and lack of adequate support systems from the Zimbabwean government. In this view, the available resource of social capital needs to be tapped into and amalgamated with other adaptation measures which rural people can easily define and own in pursuit of resilience and capacity building in the climate change discourse. Researchers on climate change adaptation in Zimbabwe should see through the essentiality of social capital on adaptation measures in rural communities. There is negligible literature on how social capital is a fundamental driver for adaptation in the Zimbabwean context and this prompts areas for further research.

## Data Availability

Transcripts from in-depth interviews and Focus Group Discussions (FDG) used in this article are available on request.
